# A Modified Coupled Enzyme Method for O-linked GlcNAc Transferase Activity Assay

**DOI:** 10.1007/s12575-009-9016-x

**Published:** 2009-12-03

**Authors:** Lianwen Zhang, Feifei Ren, Jing Li, Xiaofeng Ma, Peng Wang

**Affiliations:** 1 College of Pharmacy, Nankai University, Tianjin, 300071, People's Republic of China; 2 The State Key Laboratory of Elemento-organic Chemistry, Nankai University, Tianjin, 300071, People's Republic of China; 3 Departments of Biochemistry and Chemistry, the Ohio State University, Columbus, OH, 43210, USA

**Keywords:** O-linked GlcNAc transferase (OGT), coupled enzyme method, MS-glycosylation assay, kinetic study

## Abstract

In order to determine the activity of O-linked GlcNAc transferase (OGT), a modified coupled enzyme method was proposed. This method was based on the measurement of uridine 5'-(trihydrogen diphosphate) (UDP), a product generated in transglycosylation reaction. In the assay, UDP was coupled to the conversion of phosphoenolpyruvate to pyruvate using pyruvate kinase. Using a commercial pyruvate assay kit, the pyruvate was converted to a red terminal product, which could be photometrically measured at 570 nm or fluorometrically measured at 587 nm (*E*_m_ = 535 nm) on a microplate reader. Kinetic study of a truncated recombinant mOGT and quantitative analysis of OGT in two biological samples indicated that this method was practical and competitive for quantitative analysis of OGT.

## 1. Introduction

*O*-linked *N*-acetylglucosaminyltransferase (OGT) is an important enzyme for protein post-translational modifications. It catalyzes the transfer of O-linked GlcNAc to serine/threonine residues of a variety of substrate proteins, including nuclear pore proteins, transcription factors, and proteins implicated in diabetes and neurodegenerative disorders [1,2].

In order to determine the activity of OGT, several methods have been put forward based on detection of glycosylated products, including (1) FRET method [3], (2) MS-based method [4,5] and (3) radioactive label methods [6,7]. In contrast to other two methods, radioactive label was regularly used due to its sensitivity and competence for quantitative analysis. However, the materials used in radioactive label methods are expensive, and the methods require time-consuming steps to separate unreacted products. In addition, professional training and peculiar radiation protection are also required during the process.

Unlike the above methods, a coupled enzyme method has been contrived to assay UDP-glucuronyltransferase (EC 2.4.1.17) [8]. This method is based on the measurement of UDP, a product generated in most of transglycosylation reactions. In the assay, UDP was coupled to the conversion of NADH into NAD^+^ through pyruvate kinase and lactate dehydrogenase; the NAD^+^ was photometrically measured at 340 nm. Inconveniently, the detection wavelength is within ultraviolet range, which limits its application on high-throughput assays that could be conducted on microplate readers.

In the present work, a modified coupled enzyme method was proposed for kinetic the study of OGT and OGT amount assay in various samples. This method was based on the coupling of pyruvate-generating reaction and pyruvate assay in one system using enzyme-linked method. Kinetic study of a truncated recombinant mOGT and quantitative analysis of OGT in two biological samples proved that this method was practical and competitive for quantitative analysis of OGT.

## 2. Materials and Methods

### 2.1. Materials

UDP, UDP-GlcNAc, and GlcNAc were purchased from Merck Chemical Co. Agarose wheat germ agglutinin (WGA-agarose) (AL-1023) was purchased from Vector Laboratories Inc. PEP-K, pyruvate kinase (PK) were BBI products. Pyruvate assay kit (K609-100) was a BioVision Research Product. OGT antibody (11576-2-AP) was purchased from Proteintech Group Inc. Other antibodies were purchased from Beijing Dingguo Biotechnology, Inc. Peptide (YSDSPSTST) was synthesized in GL Biochem Ltd. (Shanghai).

### 2.2. Overexpression and Purification of a Truncated Recombinant mOGT

Human mOGT constructed in pET-32b was a gift kindly provided by Prof Hanover (NIDDK, National Institutes of Health) and was used as the template here. A truncated DNA fragment containing five TPRs and the full catalytic domain (about 654 amino acids) was obtained by PCR using primers 5'-GCGCGGATCCTTTGCT GATGCCTACT (up) and 5'-GAGAGCGGCCGCTGCTGACTCAGTGACTTCAAC AG (down), which was then constructed into pET-21a to form a recombinant plasmid with a C-terminal His-tag. The recombinant plasmid was transformed into *Escherichia coli* BL21 (DE3). The recombinant protein (C-654) was overexpressed via IPTG induction (0.1 mM) and was purified with Ni^2+^-affinity column. The purified C-654 was resolved by sodium dodecyl sulfate polyacrylamide gel electrophoresis and visualized by Coomassie Brilliant Blue (R-250) staining method. The result was further confirmed by His-tag specific Western blotting.

### 2.3. Protein Concentration Assay

Protein concentration was determined according to Coomassie brilliant blue (G-250) method [9].

### 2.4. MS-Glycosylation Assay

MS-Glycosylation assay was based on mass determination of the product. It was widely used in qualitative analysis because of its sensitivity.

The reaction mixture for a standard assay included the recombinant C-654 (0.03 mg), peptide (0.25 mM), and UDP-GlcNAc (0.5 mM) in the kit buffer (provided in the pyruvate assay kit) in a final volume of 120 μL. The control was identical to the standard assay except for the absence of the recombinant C-654. The reaction mixture was incubated at room temperature for 30 min, and the reaction was stopped by boiling the mixture for 5 min. After centrifugation at 14,000×*g* for 10 min, the supernatant was loaded on WGA-agarose. After water rinsing three to five times, glycosylated peptide was eluted with 0.5 mL 300 mM GlcNAc. The eluate was monitored on an IonSpec QFT mass spectrometer (Varian) using electron spray ionization (ESI) and using PEG1450 as the internal standard. The standard GlcNAc and C-654 were also assayed using the ESI-MS.

### 2.5. Coupled Enzyme Assay of the Recombinant C-654

Coupled enzyme assay of the recombinant C-654 was according to the following reactions:(1)

(2) $$\text{UDP}+\text{PEP}\overset{\text{PK}}{\underset{}{\to }}\text{UTP}+\text{Pyruvate}$$

(3)

There were two products in OGT-mediated transglycosylation reaction. One was the glycosylated Peptide (glyco-peptide); the other was UDP (**Eq.** 1). UDP could be coupled to the conversion of PEP to pyruvate using PK (**Eq.** 2) [8]. The pyruvate was catalyzed to its oxidative product with enzymes provided in pyruvate assay kit, and the oxidative product could react with a probe (also provided in the kit) to generate terminal product with maximum absorption at 570 nm (**Eq.** 3). The optical density at 570 nm (OD_570nm_) was measured on a microplate reader (BioRad 680). According to the manual, the terminal product could also be monitored fluorometrically at 587 nm (*E*_m_ = 535 nm).

Standard curve for pyruvate measurement was performed according to the manufacturer's protocol. To gain insight into the feasibility to perform UDP assay, varying concentrations of UDP with additional PEP (0.7 mM) and PK (2 units) were added into the reaction mixture in each well. Both pyruvate and UDP concentrations were ranged from 0.02 to 0.2 mM. The plot of UDP concentration versus OD_570nm_ was constructed, and the best-fit-line through experimental points was obtained, from which OD_570nm_ could be converted into UDP concentration (Appendix).

The coupled enzyme assay of C-654 was conducted in a standard reaction mixture consisting of C-654 (0.03 mg), PEP (0.7 mM), PK (2 U), probe (2 μL), and enzyme mix (2 μL) in a total volume of 100 μL. Varying concentrations of peptide or UDP-GlcNAc were added into the mixture in the presence of 0.5 mM UDP-GlcNAc or the peptide. The substrate concentration ranged from 0.05 to 0.5 mM. OD_570 nm_ was recorded in every 5 min from 0 to 60 min. Reaction rate was calculated according to UDP concentration and the following equation:

$$\upsilon =\frac{\left[\text{UDP}\right]}{\Delta t}$$

where *υ* denotes the reaction rate, [UDP] is the UDP concentration calculated according to the best fit line, and Δ*t* is the time span of the reaction. Kinetic parameters for both substrates were obtained using the Lineweaer–Burk method.

### 2.6. OGT Determination in Biological Samples

Enzyme concentration influence was performed under varying amount of C-654 in the reaction mixture consisting UDP-GlcNAc (0.5 mM), peptide (0.5 mM), PEP (0.7 mM), PK (2 U), probe (2 μL), and enzyme mix (2 μL) in a total volume of 100 μL. According to pre-experiment, the amount of C-654 was restricted to the range of 1–50 μg per well. Data were recorded 15 min later after the addition of C-654.

Biological sample used here was total protein prepared either from the IPTG-induced BL21 or from fresh mice brain tissue. Purified C-654 was used as a positive control. Both purified C-654 and the total protein were changed into standard OGT reaction buffer (50 mM Tris–HCl, pH 7.4, 1 mM DTT, 10 mM KCl, and 12.5 mM MgCl_2_) and condensed using a 30,000 molecular weight cutoff (MWCO) centricons (Millipore). Pre-reaction of the OGT-mediated transglycosylation was performed at room temperature for 30 min in 90 μL OGT reaction buffer consisting of UDP-GlcNAc (0.5 mM) and peptide (0.5 mM). Then, 10 μL reaction mix consisting of PEP (0.7 mM), PK (2 U), probe (2 μL), and the enzyme mix (2 μL) was added into the above solution. Fluorescence at 587 nm (*E*_x_ = 535 nm) was recorded on a Varioskan^®^ Flash multimode reader (Thermo Scientific) in every 5 min from 0 to 30 min. OGT in the samples was further validated by OGT-specific Western blotting.

All assays on microplate were performed in triplicate, and mean value was used in this work.

## 3. Results and Discussion

### 3.1. Overexpression and Purification of Recombinant C-654

**Figure 1:**
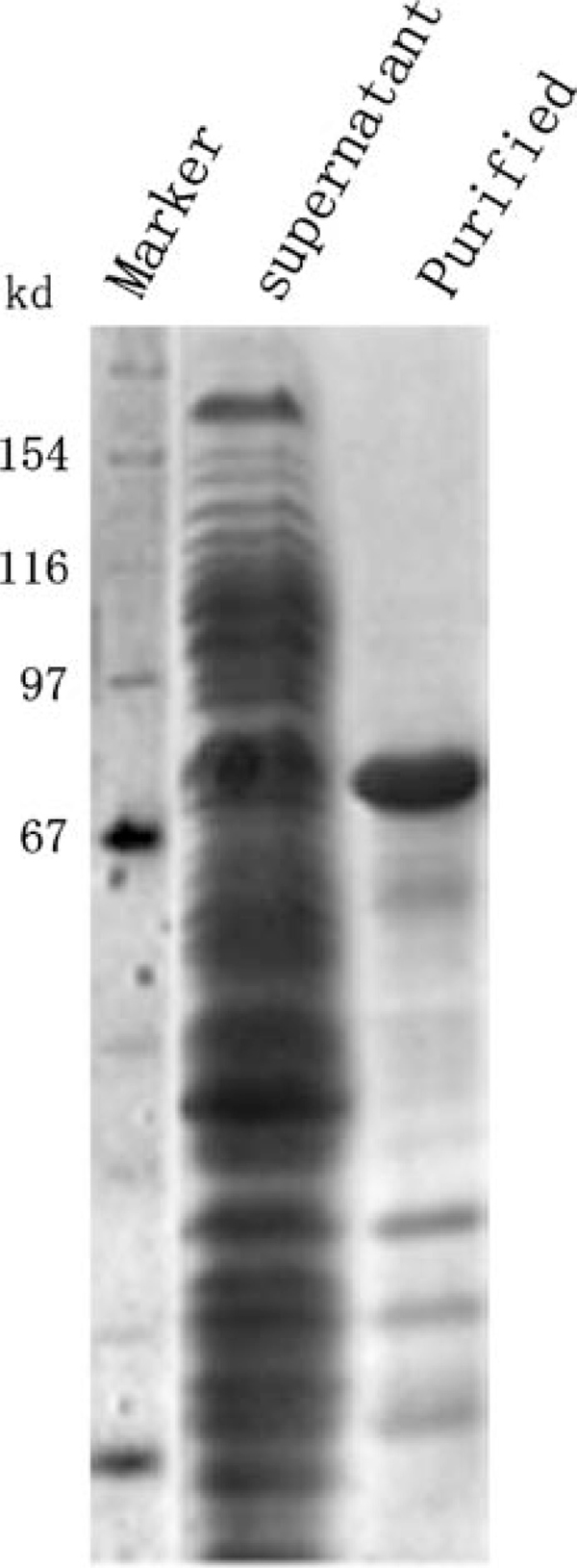
**Expression and purification of C-654**. *Lane 1* Protein standard; *lane 2* supernatant; *lane 3* purified recombinant C-654 by Ni^2+^ affinity column.

The full length and functional human mOGT was difficult to be expressed in *E. coli *[7,10]; similar result was found in our pre-experiment. In an effort to realize the soluble and functional OGT expression, the truncated mOGT recombinant (C-654) was constructed and expressed. After Ni^2+^ affinity column purification, the highly purified C-654 was obtained, which was characterized with a MW of 75 kD (the theoretical MW was 73 kD; Figure 1). C-654 was further identified by His-tag specific Western blotting (data not shown).

### 3.2. MS-Glycosylation Assay

It has been reported that the repeat times of the tetratricopeptide repeats (TPRs) is important for the OGT to carry out its transglycosylation function. A truncated recombinant mOGT containing five TPRs was able to execute the transglycosylation function in vitro but that containing two TPRs had no detectable transglycosylation function [2]. In this work, C-654 also consisted of five TPRs, but the specific amino acid sequence was different from the reported one. Therefore, we evaluated the activity of C-654 prior to its kinetic study.

To our knowledge, MS-glycosylation assay should be the best candidate method to analyze the enzyme activity qualitatively because of its sensitivity and convenience. Several MS-glycosylation methods have been used in determining glycosylated products, most of which used synthesized peptides as the glyco-acceptor [4,5]. Accordingly, we selected a widely used peptide—YSDSPSTST—in this work. Referring to Robert's work, we determined the glyco-peptide on the QFT mass spectrometer.

**Figure 2:**
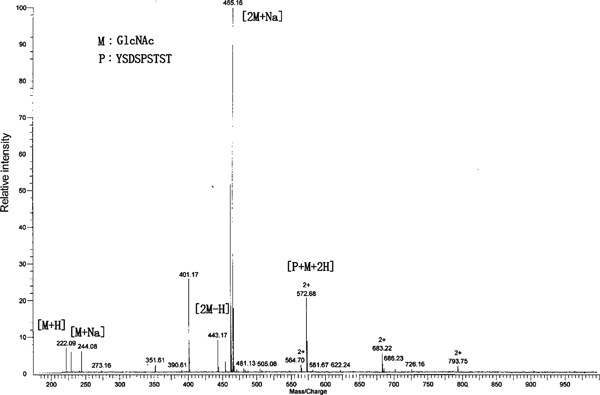
**MS-Glycosylation assay of C-654 activity**. The glyco-peptide was assayed on a QFT-ESI mass spectrometry, whose hydrogen adduct was shown as a double charged ion (*m*/*z* 573) on the mass spectrum.

The molecule weight of the glyco-peptide was 1,144 Da, whose hydrogen adducts [glyco-peptide + H]^+^ should appear at *m*/*z* 1,145 on the mass spectra. To our surprise, there was no signal appearing in the range from *m*/*z* 1,000 to 1,500 (data now shown), indicating that there was no [glyco-peptide + H]^+^ formation during the course of ionization. In the presence of C-654, a double-charged ion appeared at *m*/*z* 573 (Figure 2). While for the control and C-654 standard, no signal at *m*/*z* 573 was found (data not shown), suggesting that the double-charged ion should be assigned to a product generated from the transglycosylation reaction. According to the reaction and molecular weight of the glyco-peptide (1,144 Da), the double-charged ion could be assigned to [glyco-peptide + 2H]^2+^. It was somewhat similar to Robert's work, where they indicated that doubly charged ion might emerge on the mass spectrum instead of the singly charged ion [4]. Other ions peaking at *m*/*z* 222, 244, 443, and 465 were assigned to [GlcNAc + H]^+^, [GlcNAc + Na]^+^, [2GlcNAc + H]^+^, and [2GlcNAc + Na]^+^, respectively, according to the mass spectrum of GlcNAc and the relationship among these ions (data not shown). These results confirmed the C-654 activity in transglycosylation reaction.

### 3.3. Coupled Enzyme Assay of C-654

The study on an enzyme always focuses on its zymological properties. The routine method used for zymological property study of OGT was the radioactive label method. Although coupled enzyme method was used to assay glucuronyltransferase 30 years ago [8], recent investigations for glycotransferases still depended on radioactive label method. One of the possible reasons might be the inconvenience of the detection wavelength in the coupled enzyme method. Fortunately, we found a commercial assay kit that was clinically used to assay pyruvate in blood. Webber *et al*. [11] have used the kit to assay pyruvate amount in some species of bacteria. Considering that this kit could perform pyruvate microanalysis in complex blood samples and bacterial lysates, it should be most likely to fulfill pyruvate assay in a coupled enzyme system. Base on this hypothesis, we put forward the modified coupled enzyme method. In this method, we coupled pyruvate generating reaction and pyruvate assay in a unit system using an enzyme-linked method.

**Figure 3:**
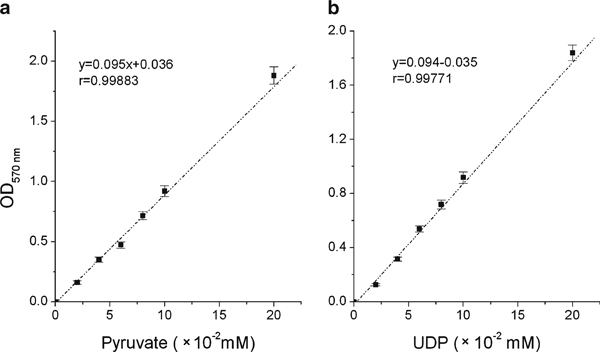
**Standard assay of pyruvate and UDP**. **a** Standard assay of pyruvate was performed according to the manufacturer's protocol. **b** Standard assay of UDP was performed in the presence of PEP (0.7 mM) and PK (2 U). Both pyruvate and UDP concentration was ranged from 0.02 to 0.2 mM. The *error bars* indicate the range of standard deviation of the experimental data.

In the standard pyruvate assay, OD_570 nm_ was directly proportional to the amount of pyruvate present in the sample (Figure 3a), suggesting that the kit works well in the experimental concentration range. We next assayed varying concentrations of UDP using the kit. It was found that OD_570 nm_ also rose linearly with increasing UDP concentration (Figure 3b), indicating that UDP could be analyzed quantitatively using the coupled enzyme method.

**Figure 4:**
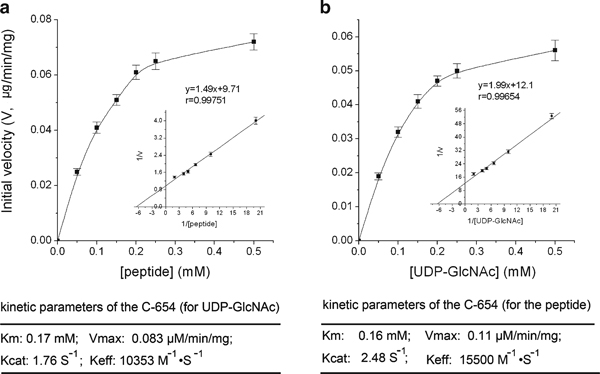
**Kinetic study of C-654**. Effect of UDP-GlcNAc or peptide concentration on C-654 activity. Assays were performed under (**a**), varying UDP-GlcNAc concentration from 0.05 to 0.5 mM while holding peptide concentration at 0.5 mM; **b** varying peptide concentration from 0.05 to 0.5 mM and holding UDP-GlcNAc concentration at 0.5 mM, respectively. The *inset figures* show double-reciprocal plots for UDP-GlcNAc and peptide, respectively. The zymological parameters of C-654 were indicated at the *bottom*. The *error bars* indicate the range of standard deviation of the experimental data.

In transglycosylation reaction performed under different substrates concentration, OD_570 nm_ increased linearly with time expanding until 30 min (data now shown), according to which 30 min was adopted in the following assays. UDP concentration could be worked out according to OD_570 nm_ and the equation: *y* = 0.094*x* - 0.035, where *y* denotes the absorbance at 570 nm and *x* denotes the UDP concentration. Next, the average reaction rate in 30 min was calculated, and the plot of reaction rate versus substrate concentration was constructed (Figure 4). It was obvious that the velocity increased linearly with mounted UDP-GlcNAc concentration from 0.05 to 0.15 mM. When the concentration was greater than 0.2 mM, the velocity increased more and more slowly. Similar result was shown for the peptide. From the double-reciprocal plot (inset figures in Figure 4), the *k*_m_ for UDP-GlcNAc and peptide was calculated to be 0.17 and 0.16 mM, respectively, indicating that C-654 had similar affinity to them.

Using the coupled enzyme method, it was easy to evaluate the C-654 activity by continuous measurement of OD_570 nm_. Kinetic study of C-654 indicated that the *k*_m_ for UDP-GlcNAc (0.17 mM) was in the similar level to that of the reported 5TPR-containing OGT truncates (60 μM) [2]. The discrepancy might be due to the difference of OGT variant and substrate.

### 3.4. OGT Determination in Biological Samples

**Figure 5:**
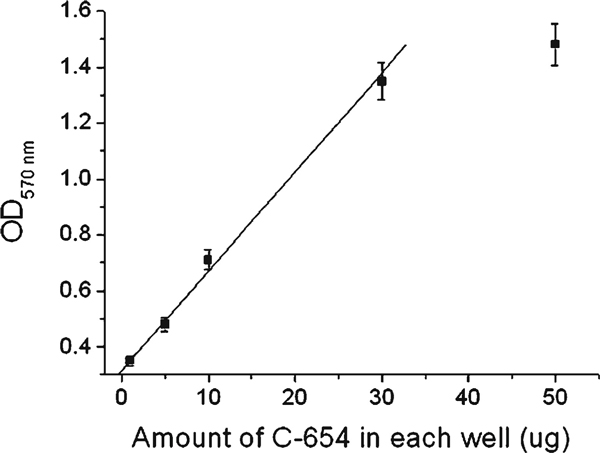
**Linear relationship between C-654 amount and OD_570 nm_**. Assays were performed under varying C-654 amount from 1 to 50 μg per well in the standard reaction mixture as indicated in "Materials and Methods." The *error bars* indicate the range of standard deviation of the experimental data.

To apply this coupled enzyme method in determining the amount of functional OGT, we evaluated the linear relationship between the amount of C-654 and OD_570nm_. OD_570nm_ was linearly increased with the increasing amount of C-654 in the range from 1 to 30 μg per well (Figure 5). The nonlinearly increase in OD_570nm_ at a higher concentration (50 μg per well) should be due to overloading of the enzyme. We found that the more the enzyme, the sooner OD_570nm_ reaches the climax. In fact, the mixture solution turned red soon after the addition of the last component (purified C-654) at the high concentration condition.

**Figure 6:**
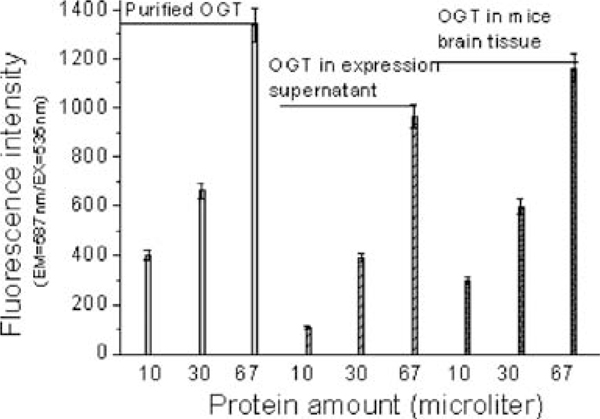
**OGT assay in biological samples**. Assays were performed under varying OGT amount in different samples. The data were collected 20 min after starting. Pre-reaction of the OGT mediated transglycosylation was performed at room temperature for 30 min in 90 μL OGT reaction buffer consisting of UDP-GlcNAc (0.5 mM) and peptide (0.5 mM). Then, 10 μL reaction mix consisting of PEP (0.7 mM), PK (2 U), probe (2 μL), and the enzyme mix (2 μL) was added into the above solution. Fluorescence at 587 nm (*E*_x_ = 535 nm) was recorded in 15 min. (1) Purified C-654 (*light gray*); (2) total protein extracted from sonicated C-654 expressing BL21 solution (*gray*); (3) total protein extracted from mice brain tissue (*heavy gray*). The *error bars* indicate the range of standard deviation of the experimental data.

The above results indicated that the coupled enzyme method is feasible to determine the amount of purified OGT. It would be more valuable if it could be performed to determine OGT amount in complex biological samples. To address this question, we prepared two kinds of biological samples; one was the coarse protein solution extracted from the IPTG-induced C-654-expressing BL21, the other was total protein solution extracted from the fresh mice brain tissue, which was proved to be one of the tissues rich in OGT [12,13]. To avoid failure caused by little content of OGT, both samples were condensed by ultrafiltration using a 30,000-MWCO centricons. In addition, we performed the pre-reaction of the OGT-mediated transglycosylation and used the sensitive fluorometric assay in the experiment. For both biological samples, the rising amount of OGT gave increasing fluorescence intensity, as for the purified C-654 (Figure 6), which was proven by OGT-specific Western blotting (data not shown). In our pre-experiment, we found that imidazole (50 mM) could diminish the fluorescence intensity, especially in the first 10 min. Further investigation indicated that the diminishment was caused by the inhibition of the enzyme mix of the kit. Therefore, imidazole should be wiped off to assay OGT amount, provided that the OGT was purified using the Ni affinity chromatography method. Some small molecules such as UDP or ADP should also be wiped off in complex biological samples, in that both these substrates would react with PEP and generate pyruvate and increase the fluorescent intensity. In this work, the small molecules were wiped off by buffer changing.

In summary, the modified coupled enzyme method was a valuable method for OGT activity assay, especially for high throughput assay. In contrast with the radioactive label method, this method had the superiority to assay OGT in a mixture solution without further separation. Theoretically, this method might be extended to evaluate other glycosyltransferase-mediated transglycosylation reactions only if the reaction had a UDP product. However, for some biological samples, some disturbed molecules such as UDP and ADP should be drastically wiped off. Confined by the pyruvate assay kit, the detection limit of the modified coupled enzyme method was at a micromolar concentration level, which might be a limitation for its application in assaying low efficient glycosyltransferases in contrast to radioactive label method.

## Abbreviations

WGA-Agarose: Agarose wheat germ agglutinin; FRET: Fluorescence resonance energy transfer; PEP-K: Mono-potassium phosphoenolpyruvate; OGT: O-linked *N*-acetylglucosaminyltransferase; PK: Pyruvate kinase; TPR: Tetratricopeptide repeat; UDP: Uridine 5'-(trihydrogen diphosphate); IPTG: Isopropyl β-D-thiogalactoside

## Appendix

### Protocol

#### Protocol I: Assay for Purified Recombinant OGT (with a His-tag)

Reagents:

• Ni affinity column

• Binding buffer: 50 mM PBS (pH 7.4), 0.5 M NaCl, 10 mM imidazole, 10% glycerol, 1% Tween-20, 1% TritonX100

• Washing buffer: 50 mM PBS (pH 7.4), 0.5 M NaCl, 50 mM imidazole, 10% glycerol, 1% Tween-20, 1% TritonX100

• Elution buffer: 50 mM PBS (pH 7.4), 0.5 M NaCl, 250 mM imidazole, 10% glycerol, 1% Tween-20, 1% TritonX100

• Reaction buffer: 50 mM Tris–HCl, pH 7.4, 1 mM DTT, 10 mM KCl and 12.5 mM MgCl_2_

• Centricons: 30,000 MWCO

• PEP: Mono-Potassium phosphoenolpyruvate (35 mM)

• Pyruvate kinase: (50 U/μL)

• UDP

• Peptide: YSDSPSTST

• Pyruvate assay kit

OGT expression and purification:

Overexpress the recombinant OGT with a His-tag in *E. coli* BL21 (DE3) via IPTG induction (0.1–1.0 mM) and purify OGT using Ni^2+^-affinity column according to the manufactory's manual. Change OGT in elution buffer into reaction buffer and condensed by ultrafiltration.

OGT assay using the coupled enzyme method:

1. Standard curve preparations: Dilute the UDP Standard to 1 nmol/μl; add 0, 2, 4, 6, 8, 10 μl into a series of standards wells. Adjust volume to 80 μl/well with pyruvate assay buffer to generate 0, 2, 4, 6, 8, and 10 nmol/well of the pyruvate standard for the colorimetric assay (0, 0.2, 0.4, 0.6, 0.8, and 1.0 nmol/well for the fluorometric assay).

2. Sample preparations: Prepare test samples in 80 μl/well with reaction buffer in a 96-well plate, include UDP-GlcNAc (0.5 mM), peptide (0.5 mM), and varying amount of OGT.

3. Reaction mix preparation: Mix enough reagents for the number of assays performed. For each well, prepare a total 20 μl reaction mix containing the following components. Mix well before use:

Twelve microliters reaction buffer

Two microliters PEP (0.7 mM)

Two microliters pyruvate kinase (2 units)

Two microliters pyruvate probe

Two microliters enzyme mix

4. Add 20 μl of the reaction mix to each well containing the pyruvate standard or test samples, mix well.

5. Incubate the reaction for 30 min at room temperature, protect from light.

6. Measure OD_570 nm_ for colorimetric assay or fluorescence at *E*_x_/*E*_m_ = 535/587 nm in a microplate reader.

7. Calculation

Attention:

♦ Imidazole should be drastically wiped off from the purified OGT.

#### Protocal II: Assay for OGT in Biological Samples

Reagents:

• Total protein extraction kit

• Reaction buffer: 50 mM Tris–HCl, pH 7.4, 1 mM DTT, 10 mM KCl and 12.5 mM MgCl_2_

• Centricons: 30,000 MWCO

• PEP: Mono-potassium phosphoenolpyruvate (35 mM)

• Pyruvate kinase: (50 U μL)

• UDP

• Peptide: YSDSPSTST

• Pyruvate assay kit

Total protein extraction form mice brain tissue:

Kill the mice by decapitation and take out the brain tissue quickly. Quantify the tissue and extract the total protein in a homogenizer as described in the kit manual. Additional OGA inhibitor (PUGNAc, 1 μM) should be added before the homogenization. Change total protein in lysis buffer into reaction buffer and condensed by ultrafiltration.

OGT assay using the coupled enzyme method:

1. Standard curve preparations: Dilute the UDP Standard to 1 nmol/μl; add 0, 2, 4, 6, 8, 10 μl into a series of standards wells. Adjust volume to 80 μl/well with pyruvate assay buffer to generate 0, 2, 4, 6, 8, and 10 nmol/well of the pyruvate standard for the colorimetric assay (0, 0.2, 0.4, 0.6, 0.8, 1.0 nmol/well for the fluorometric assay).

2. Sample preparations: Prepare test samples in 80 μl/well with reaction buffer in a 96-well plate, include UDP-GlcNAc (0.5 mM), peptide (0.5 mM), and varying amount of total proteins extracted from brain tissues.

3. Reaction mix preparation: Mix enough reagents for the number of assays performed. For each well, prepare a total 20 μl reaction mix containing the following components. Mix well before use:

Twelve microliters reaction buffer

Two microliters PEP (0.7 mM)

Two microliters pyruvate kinase (2 U)

Two microliters pyruvate probe

Two microliters enzyme mix

4. Add 20 μl of the reaction mix to each well containing the pyruvate standard or test samples, mix well.

5. Incubate the reaction for 30 min at room temperature, protect from light.

6. Measure OD _570 nm_ for colorimetric assay or fluorescence at *E*_x_/*E*_m_ = 535/587 nm in a microplate reader.

7. Calculation

Attention:

♦ Small molecules such as UDP and ADP should be drastically wiped off from the total protein.

♦ Total protein extraction form mice brain tissue must be proceeded on ice
